# Post-prandial amino acid and leucine responses to serum albumin concentrate compared to whey protein concentrate in healthy subjects

**DOI:** 10.1186/1550-2783-11-S1-P51

**Published:** 2014-12-01

**Authors:** Eric M Weaver, John A Rathmacher, Christopher J Detzel, Paul Khoo, Rick Sharp

**Affiliations:** 1 Proliant Health, Ankeny, Iowa, USA; 2 Metabolic Technologies Inc., Ames, Iowa, USA; 3 Iowa State University, Ames, Iowa, USA

## Background

Supplemental protein sources vary in composition in regards to size, structure, and amino acid composition of the varying individual proteins. Serum albumin concentrate (SA) is a new protein source extracted from edible-grade bovine plasma, which was recently made available on commercial scale. As compared to the low MW proteins in whey protein concentrate (WPC), SA consists of higher MW protein constituents such as albumin (66kD, >60%) and globulin proteins (130 kD, >10%), which contain a high quality amino acid composition. These differences in the protein profile of SA may both delay and sustain amino acid absorption in comparison to WPC. The objective of this study was to determine the post-prandial amino acid response to oral ingestion of SA and to compare the response to that of WPC.

## Methods

Eight healthy, 20-30 year old, male (n=4, 80.3 kg BW) and female (n=4, 63.7 kg BW) volunteers participated in an open label, clinical study utilizing a cross-over design (Clinicaltrials.gov: NCT01643265). The volunteers received each of the two protein sources in a random order with a 7 d washout period between treatments. After a 12 h overnight fast, 7 mL of blood was collected from a catheter inserted into a forearm vein. Baseline amino acid values at 30 and 15 min were determined prior to administration of the experimental protein sources. Twenty grams of either WPC or SA was provided in 335 mL of water. Subsequent blood samples were collected at 15, 30, 45, 60, 90, 120, 180, and 210 min after ingestion for analysis of individual amino acid concentrations. An EZ:faastTM amino acid analysis kit was used for sample pretreatment and chromatographic separation was conducted using a Agilent 6460 triple quadrupole LC/MS system. Data were analyzed using repeated measures and General Linear Models procedures from SAS (Version 9.2) including treatment, gender, and test period as independent variables in the statistical model. Significance is reported as P<0.05 and P<0.01. Consent to publish the results was obtained from all participants.

## Results

Total plasma amino acid concentration at baseline was found to be higher (P<0.01) at baseline in male subjects (2,633 nmoles/mL) compared to female subjects (2,153 nmoles/mL). In contrast, plasma leucine concentration was found to be higher (P<0.05) in female subjects (156 nmoles/mL) compared to male subjects (103 nmoles/mL). Rises in post-prandial individual amino acid concentration were observed for both WPC and SA (P<0.01). The rises reflect the amino acid pattern of the protein source ingested. The ingestion of whey protein concentrate increased plasma total amino acid concentration 30 min post-prandially compared to SA (P<0.05) by 10%, without differences in leucine. In contrast, the ingestion of SA promoted 14% higher leucine concentrations 120 min post-prandially than did WPC (P<0.05).

## Conclusion

Individual post-prandial amino acids reflect the amino acid pattern and characteristics of the protein source ingested. The digestion and absorption of the protein and amino acid composition of serum albumin concentrate (SA) results in an increase in total plasma amino acids, including leucine. WPC, comprised of low molecular weight proteins,elevates the post-prandial amino acid response rapidly but SA ingestion, comprised of proteins with high molecular weights, maintains leucine concentrations over a longer time after ingestion.

